# Overexpression of *RpKTI2* from *Robinia pseudoacacia* Affects the Photosynthetic Physiology and Endogenous Hormones of Tobacco

**DOI:** 10.3390/plants13131867

**Published:** 2024-07-06

**Authors:** Jian Zhou, Pengxiang Die, Songyan Zhang, Xiaoya Han, Chenguang Wang, Peipei Wang

**Affiliations:** 1 School of Horticulture and Landscape Architecture, Henan Institute of Science and Technology, Xinxiang 453003, China; 17839577676@163.com (P.D.); zhangsy616@163.com (S.Z.); xiaoyahan2024@163.com (X.H.); 13781670739@163.com (C.W.); 16638509593@163.com (P.W.); 2 Henan Province Engineering Center of Horticulture Plant Resource Utilization and Germplasm Enhancement, Xinxiang 453003, China

**Keywords:** *Agrobacterium*-mediated transformation, chlorophyll, gene expression, *Nicotiana tabacum*, photosynthetic characteristics, stomata

## Abstract

Kunitz trypsin inhibitor genes play important roles in stress resistance. In this study, we investigated *RpKTI2* cloned from *Robinia pseudoacacia* and its effect on tobacco. *RpKTI2* was introduced into the tobacco cultivar NC89 using *Agrobacterium*-mediated transformation. Six *RpKTI2*-overexpressing lines were obtained. Transgenic and wild-type tobacco plants were then compared for photosynthetic characteristics and endogenous hormone levels. Transgenic tobacco showed minor changes in chlorophyll content, fluorescence, and photosynthetic functions. However, the maximum photochemical efficiency (*F*_v_/*F*_m_) increased significantly while intercellular CO_2_ concentration (*C*_i_) decreased significantly. Stomatal size and hormone content (indole-3-acetic acid, zeatin riboside, gibberellin, and indole-3-propionic acid) were reduced, while brassinosteroid content increased. Random forest regression revealed that *RpKTI2* overexpression had the biggest impact on carotenoid content, initial fluorescence, *C*_i_, stomatal area, and indole-3-acetic acid. Overall, *RpKTI2* overexpression minimally affected chlorophyll synthesis and photosynthetic system characteristics but influenced stomatal development and likely enhanced the antioxidant capacity of tobacco. These findings provide a basis for future in-depth research on *RpKTI2*.

## 1. Introduction

Trypsin inhibitors (TI), also known as antitrypsins, are serine protease inhibitors [1] in the protease inhibitor family. Soybean TIs typically have the highest activity among the various TIs. Kunitz TI (KTI) genes contain a unique Kunitz domain and belong to a gene family that specifically inhibits proteases [2]. KTIs are widely distributed in plants such as *Glycine max*, *Adenanthera pavonina*, *Carica papaya*, *Vigna umbellata*, *Sophora alopecuroides*, *Alocasia macrorrhizos*, *Arachis hypogaea*, *Brassica integrifolia*, *Erythrina variegata*, and *Populus* [1,2]. They participate in protein storage, regulation of endogenous protease activity, and plant defense and development [3].

There is an extensive body of research on KTIs. As significant plant stress resistance genes, KTIs influence responses to biotic [4,5] and abiotic stresses [6,7]. A study on the effects of *AtKTI4* and *AtKTI5* silencing by T-DNA insertion in *Arabidopsis* showed that the independent KTI-silenced clones were more susceptible to *Tetranychus urticae* than wild-type (WT) plants [4]. Another study that introduced soybean *TI* genes to *Arabidopsis* reported that *GmKTI7* clones exhibited significantly increased resistance to *Helicoverpa zea*. Furthermore, overexpression plants showed significantly reduced leaf defoliation during infestation but did not differ significantly from WT plants in terms of growth and productivity under normal conditions [5]. Similarly, soybean *GmTI* hindered the development of *Heterodera glycines* [8], and tobacco *NtTI* conferred resistance against *Prodenia litura* and *Helicoverpa armigera* [9]. Other researchers observed that *Alternaria alternata* infection in *Nicotiana attenuata* leaves led to a high degree of *NaKTI2* induction [10], thereby enhancing the disease resistance of plants. Programmed cell death (PCD) is a core regulatory process in plant responses to pathogens, and KTIs can act as cofactors to regulate PCD. For instance, the *AtKTI1* expression in *Arabidopsis* negatively regulated lesion development triggered by the PCD-eliciting fungal toxin fumonisin B1 or the avirulent bacterial pathogen *Pseudomonas syringae* pv. tomato DC3000 carrying avrB [11].

*KTI* genes are also prone to induction by abiotic stresses. For instance, *PtKTI12A* and *PtKTI12B* in poplar are induced by heat and drought stresses and shown to participate in elongator complex formation and tRNA wobble uridine modification, which lead to enhanced stress resistance through effects on metabolites and signaling pathways [7]. When *Arabidopsis* was transformed with the *KTI* genes of *Cassia obtusifolia* (*COTI1* and *COTI2*), the transcription levels of both genes were upregulated under conditions of salt and drought stress, and the transgenic lines exhibited significant enhancement of salt and drought tolerance [12]. Another study reported that *ClKTI* in *Curcuma longa* was upregulated via the regulation of methyl jasmonate, with the roots and stems manifesting the most significant changes [13]. Upon exposure to copper stress, *PdKTI3* in *Populus deltoides* was highly upregulated, and the copper tolerance of plants was enhanced through copper chelation [14].

The black locust (*Robinia pseudoacacia*) is a deciduous tree species belonging to the Fabaceae. It exhibits strong tolerance to drought, infertile soils, and heavy metal pollution (lead and cadmium) and is an excellent tree species for environmental restoration [15]. Our research group identified a high-lead-concentration-responsive gene, *RpKTI2,* through transcriptome sequencing of *R. pseudoacacia* under lead stress. Subsequently, we cloned *RpKTI2* from black locust. The 796 bp gene (Table S1) includes a 633 bp coding sequence (Table S2) that encodes 210 amino acids (Table S3) [16,17]. The relative molecular weight of KTI2 is 22.8 kDa [17]; however, little is known about its regulatory functions.

To address this knowledge gap, in the present study, we introduced *RpKTI2* into tobacco via *Agrobacterium*-mediated transformation. Tobacco was chosen because of its large leaves and biomass, which facilitate the measurement of relevant physiological and biochemical indicators to accurately study gene function. Indicators related to photosynthetic physiology and endogenous hormones were measured in transgenic plants to investigate the influence and regulation of *RpKTI2* overexpression on the physiological characteristics of tobacco. The aim of our study was to provide a basis for future in-depth research on *RpKTI2*.

## 2. Results

### 2.1. Cultivation and Identification of RpKTI2-Expressing Transgenic Tobacco Plants

**Figure 1:**
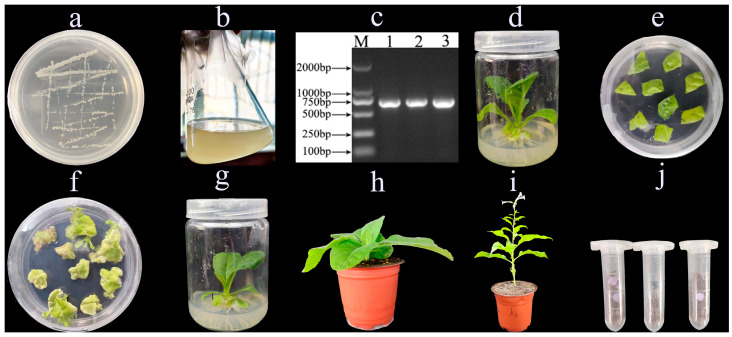
*RpKTI2* transformed into *Nicotiana tabacum* ‘NC89’: (**a**) plate coating of *Agrobacterium*; (**b**) shaking; (**c**) PCR assay of *Agrobacterium*; (**d**) sterile seedling culture; (**e**) imbibition co-culture; (**f**) bud differentiation on screening culture medium; (**g**) rooting culture; (**h**) domestication; (**i**) flowering; and (**j**) seed harvesting.

**Figure 2:**
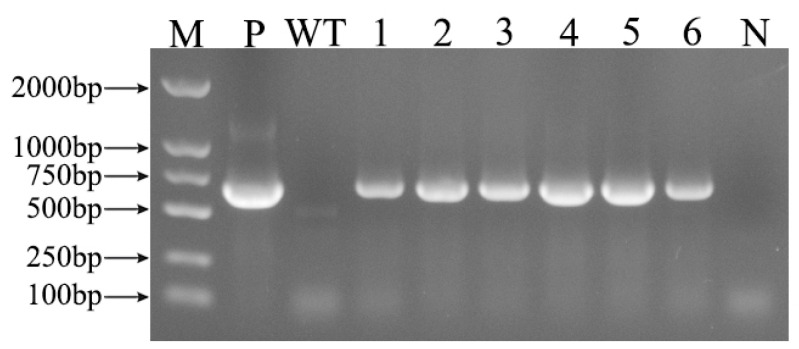
PCR detection of *Nicotiana tabacum* transgenic lines, M: DNA marker DL 2000; P: positive plasmid control; N: negative control; WT: wild-type plants; 1~6: positive lines K1, K2, K3, K4, K5, and K8.

**Figure 3:**
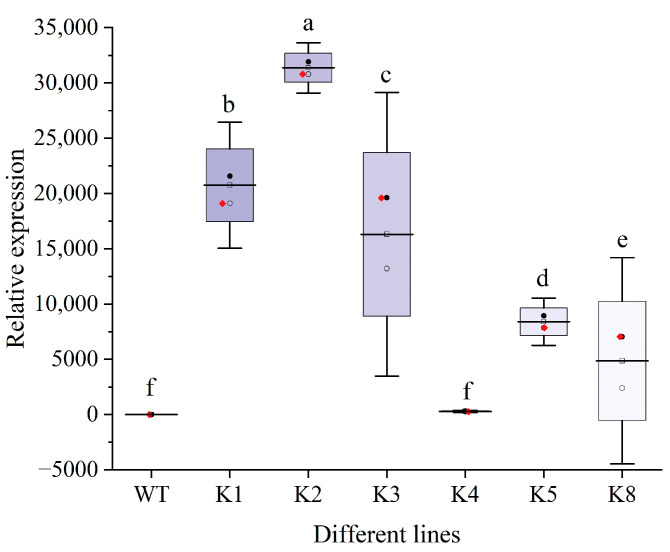
RT-qPCR detection of transgenic lines. WT: wild-type plants; K1, K2, K3, K4, K5, K8: transgenic *RpKTI2*-positive lines K1, K2, K3, K4, K5, and K8. The error line in the figure represents the standard deviation (*n* = 3), and the different lowercase letters represent the significant difference between the lines (*p* < 0.05).

Tobacco leaves were infected with a pBI121-*RpKTI2 Agrobacterium* solution (Figure 1a–e). Resistant shoots (Figure 1f) were obtained after 4–6 weeks of selective culturing. Subsequently, rooting (Figure 1g) and domestication (Figure 1h) were performed until the T0 generation seeds were obtained (Figure 1i,j). The seeds were sown onto the Kan screening medium for cultivating T1 generation transgenic plants. Upon the emergence of four true leaves, PCR testing was performed on the tobacco plants. Bright bands were present at Positions 1–6 (positive transgenic lines), whereas WT plants exhibited an absence of DNA amplification (Figure 2). This indicated the successful integration of *RpKTI2* into the genome of the corresponding transgenic lines, which were named K1, K2, K3, K4, K5, and K8. The positive transgenic tobacco plants were then subjected to RT-qPCR testing. Compared with WT plants, the six transgenic lines exhibited significantly upregulated *RpKTI2* expression. Expression levels were the highest in K1 and K2 and lowest in K4 (Figure 3).

### 2.2. Changes in RpKTI2 Levels in Transgenic Tobacco

**Figure 4:**
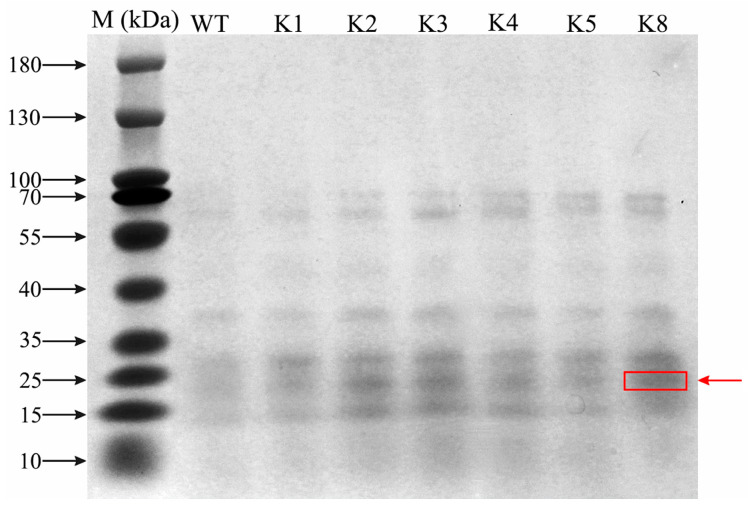
Protein SDS-PAGE analysis of transgenic tobacco lines. WT: wild-type plants; K1, K2, K3, K4, K5, K8: transgenic RpKTI2-positive lines K1, K2, K3, K4, K5, and K8. The red box and red arrow indicate the KTI2 band.

**Figure 5:**
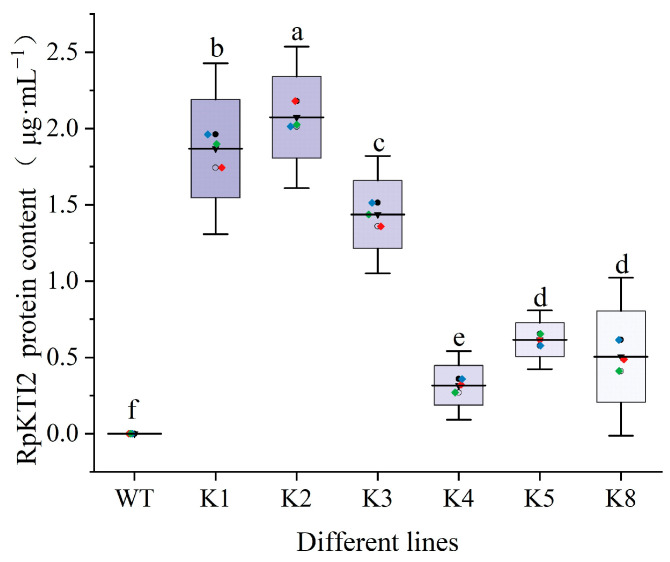
Contents of KTI2 in transgenic Nicotiana tabacum leaves. WT: wild-type plants; K1, K2, K3, K4, K5, K8: transgenic RpKTI2-positive lines K1, K2, K3, K4, K5, and K8. The error line in the figure represents the standard deviation (*n* = 3), and different lowercase letters indicate significant differences between the lines (*p* < 0.05).

SDS-PAGE electrophoresis analysis was performed on *RpKTI2*-overexpressing tobacco. Clear target protein bands were observed for the K1, K2, K3, K4, K5, and K8 lines but absent for WT (Figure 4). Quantitative detection revealed that RpKTI2 was significantly upregulated in the six transgenic lines (*p* < 0.05) compared with that in WT plants, which was consistent with the trend of *RpKTI2* expression. The K2 line had the highest RpKTI2 content. There was no significant difference (*p* > 0.05) in RpKTI2 levels between the K5 and K8 lines (Figure 5).

### 2.3. Changes of Chlorophyll Content in RpKTI2-Overexpressing Tobacco

**Figure 6:**
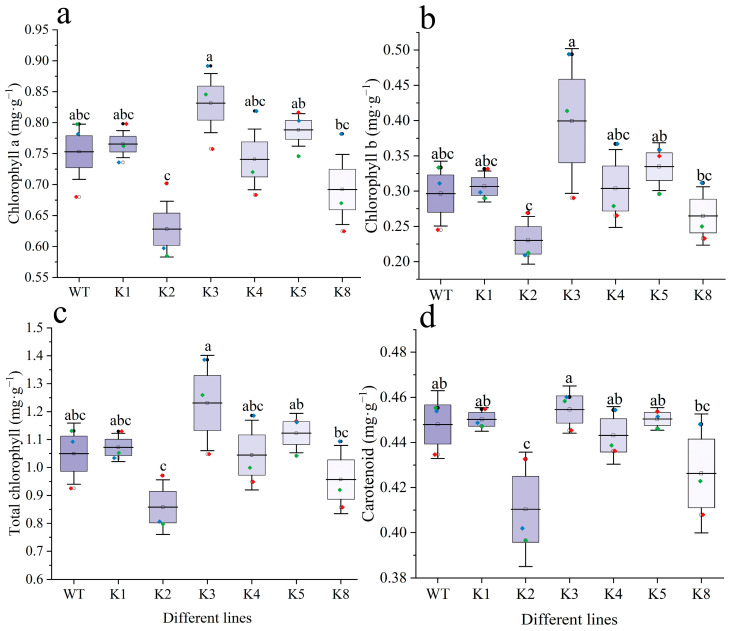
Chlorophyll and carotenoid content in transgenic and WT *Nicotiana tabacum*: (**a**) chlorophyll a content, (**b**) chlorophyll b content, (**c**) total chlorophyll content, and (**d**) carotenoid content. WT: wild-type plants; K1, K2, K3, K4, K5, K8: transgenic *RpKTI2*-positive lines K1, K2, K3, K4, K5, and K8. The error line in the figure represents the standard deviation (*n* = 3), and the different lowercase letters represent significant differences between lines (*p* < 0.05).

The Chla, Chlb, and Chla + b contents did not differ significantly between the transgenic and WT plants (*p* > 0.05) (Figure 6a–c). K3 showed the highest values for all three parameters, whereas K2 exhibited the lowest values. The carotenoid content of all transgenic lines other than K2 did not differ significantly from that of WT plants, with K3 having the highest content. The carotenoid content of K2 was significantly lower than that of WT (*p* < 0.05) (Figure 6d).

### 2.4. Changes of Chlorophyll Fluorescence Parameters in RpKTI2-Overexpressing Tobacco

**Figure 7:**
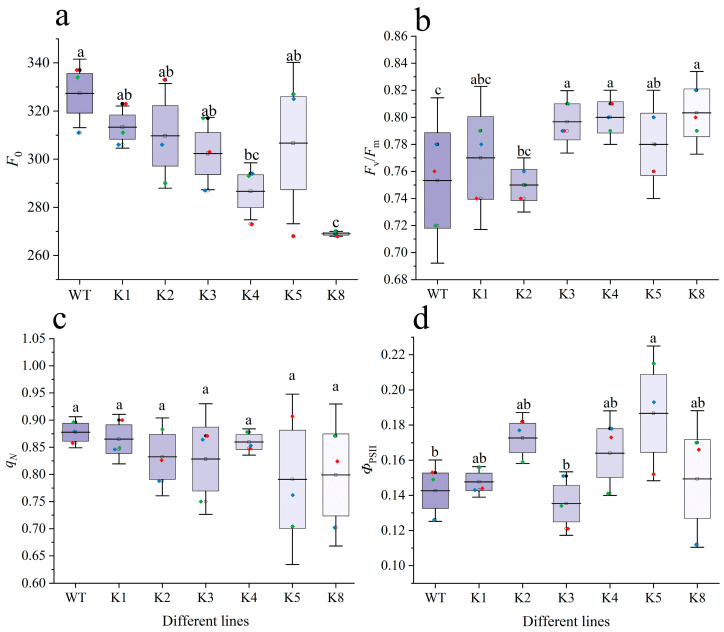
Characteristics of chlorophyll fluorescence in transgenic and WT *Nicotiana tabacum*: (**a**) initial fluorescence (*F*_0_), (**b**) maximum photochemical efficiency (*F*_v_/*F*_m_), (**c**) non-photochemical quenching coefficient (*q*_N_), and (**d**) actual photochemical quantum efficiency (*Φ*_PSII_). WT: wild-type plants; K1, K2, K3, K4, K5, K8: transgenic *RpKTI2*-positive lines K1, K2, K3, K4, K5, and K8. The error line in the figure represents the standard deviation (*n* = 4), and different lowercase letters indicate significant differences between the lines (*p* < 0.05).

*F*_0_ decreased progressively in the transgenic tobacco lines (Figure 7a). However, only K4 and K8 had values that were significantly lower than that of WT (*p* < 0.05). By contrast, *F*_v_/*F*_m_ showed a progressive increase in the transgenic lines. Except for K1 and K2, all other lines exhibited significantly higher values than WT (*p* < 0.05), with K8 having the highest value (Figure 7b). *q_N_* values of the transgenic tobacco plants showed little change and were relatively stable; differences with WT were not statistically significant (*p* > 0.05) (Figure 7c). All transgenic lines except for K3 had a higher *Φ*_PSII_ than WT, but the difference with WT was only statistically significant in K5 (*p* < 0.05) (Figure 7d).

### 2.5. Photosynthetic Function of RpKTI2-Overexpressing Tobacco

**Figure 8:**
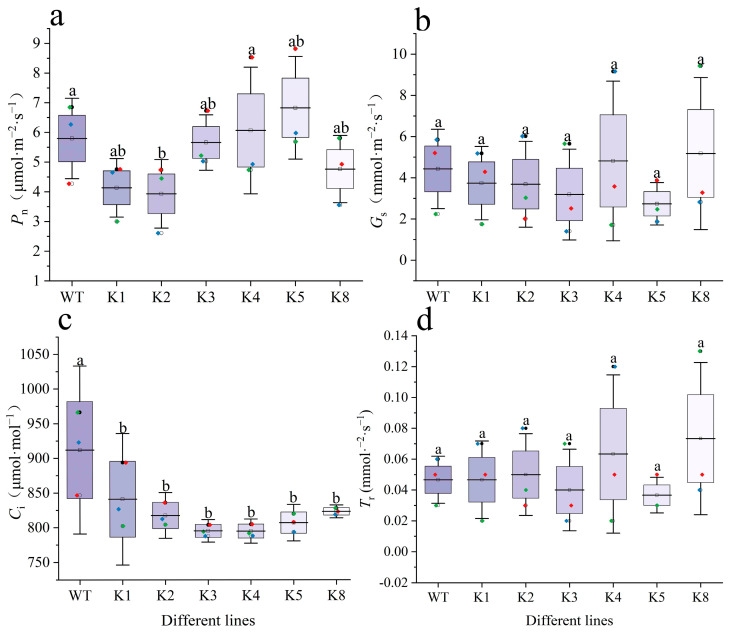
Variations of photosynthetic function of transgenic *Nicotiana tabacum* and WT: (**a**) photosynthetic rate (*P*_n_), (**b**) stomatal conductance (*G*_s_), (**c**) intercellular CO_2_ concentration (*C*_i_), and (**d**) transpiration rate (*T*_r_). WT: wild-type plants; K1, K2, K3, K4, K5, K8: transgenic *RpKTI2*-positive lines K1, K2, K3, K4, K5, and K8. The error line in the figure represents the standard deviation (*n* = 4), and the different lowercase letters indicate significant differences between the lines (*p* < 0.05).

All transgenic lines other than K4 had a lower *P*_n_ than that of the WT, but the difference was only significant for K2 (*p* < 0.05) (Figure 8a). Similarly, the *C*_i_ values of all transgenic tobacco lines were significantly lower than that of WT (*p* < 0.05), with K2 having the highest value among all transgenic lines (Figure 8c). Transgenic tobacco exhibited similar patterns and relatively varied changes in *G*_s_ and *T*_r_, but differences with WT were not statistically significant (*p* > 0.05). The highest values were observed in K4 and K8 (Figure 8b,d).

### 2.6. Observation of Stomatal Morphology in Leaves of RpKTI2-Overexpressing Tobacco

**Figure 9:**
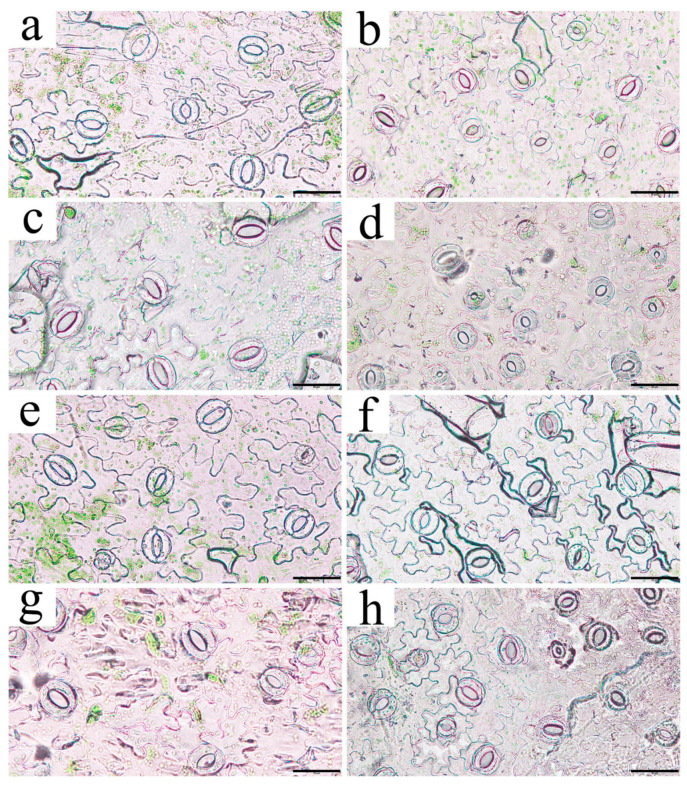
Stomatal phenotype of leaf epidermis of transgenic *Nicotiana tabacum*: (**a**,**e**) WT; (**b**–**d**, **f**–**h**) positive lines K1, K2, K3, K4, K5, and K8, respectively. Bars = 50 μm. Note: The reasons for the color difference are as follows: 1. Using transparent tape to slowly peel off the lower epidermis of the leaves to observe the stoma, and the thickness of the peeled epidermis tissue may vary among different tobacco lines. 2. There are differences in the brightness adjusted by the microscope light source when observing stomas in the leaves of different tobacco lines.

The stomatal length, width, and area were similar among transgenic tobacco lines (Figure 9 and Table 1). The values of these three indicators were lower in all transgenic lines except for K2 when compared to WT values. K2 had the highest values for all three indicators, but the differences with WT were not statistically significant (*p* > 0.05); K3 had the lowest values, significantly different from those of WT (*p* < 0.05). Changes in stomatal density were opposite to those of the other indicators. With the exceptions of K2 and K5, all positive transgenic lines had higher stomatal density than the WT. K2 and K1 had the lowest and highest stomatal density values, respectively, which were both significantly different from that of WT (*p* < 0.05).

### 2.7. Endogenous Hormone Content of RpKTI2-Overexpressing Tobacco Leaves

**Figure 10:**
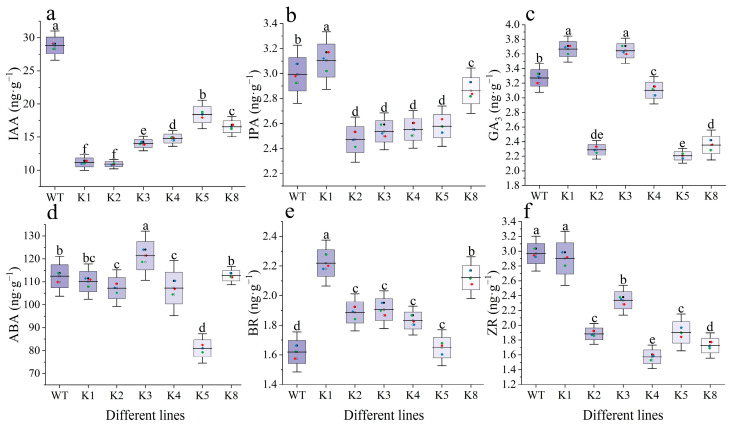
Effect of *RpKTI2* overexpression on endogenous hormone contents in *Nicotiana tabacum* leaves: (**a**) indoleacetic acid (IAA), (**b**) indolepropionic acid (IPA), (**c**) gibberellins (GA3), (**d**) abscisic acid (ABA), (**e**) brassinosteroid (BR), and (**f**) zeaxanthin riboside (ZR). WT: wild-type plants; K1, K2, K3, K4, K5, K8: transgenic *RpKTI2*-positive lines K1, K2, K3, K4, K5, and K8. The error line represents the standard deviation (*n* = 3), and the different lowercase letters indicate significant differences between the lines (*p* < 0.05).

The IAA contents of transgenic tobacco lines were significantly lower than that of WT (*p* < 0.05), with K1 and K2 having the lowest values (Figure 10a). Changes in the IPA and ZR contents were similar. Except for K1, all other transgenic lines had significantly lower IPA and ZR contents than that of the WT (*p* < 0.05). K1 had a significantly higher IPA content than that of the WT (*p* < 0.05) but its ZR content was not significantly different (*p* > 0.05) (Figure 10b,f).

Contrary to the changes in IAA content, the BR content of transgenic tobacco lines was higher than that of WT. The difference with WT was significant for all transgenic lines other than K5 (*p* > 0.05) (Figure 10e). The GA3 contents of K1 and K3 were significantly higher than those of WT (*p* < 0.05), whereas all other transgenic lines exhibited significantly lower contents than those of WT (*p* < 0.05). Changes in abscisic acid (ABA) contents were more varied. Compared with the WT, K3 had a higher ABA content whereas K2, K4, and K5 had lower contents, with all differences being statistically significant (*p* < 0.05). The ABA contents of all other transgenic lines were not significantly different from that of the WT.

### 2.8. PCA and Random Forest Regression Analysis of Various Indicators in RpKTI2-Overexpressing Tobacco

**Figure 11:**
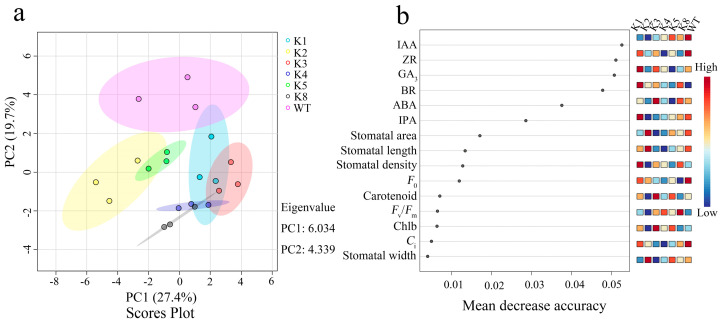
(**a**) Score plot of PCA analysis and (**b**) random forest regression analysis for each index of transgenic *Nicotiana tabacum*.

PCA was performed to further evaluate the influence of *RpKTI2* overexpression on the physiological characteristics of tobacco. PC1 and PC2 accounted for 27.4% and 19.7% of the total variance, respectively (Figure 11a). WT was obviously separated from the six transgenic lines along the PC2 axis. Among the transgenic lines, K2 and K5 were clustered and distinctly separated from the other positive lines (Figure 11a), which was similar to the clustering heatmap results (Figure S1a).

Random forest regression analysis revealed that the average accuracy values of endogenous hormones were significantly higher than those of other indicators, with IAA, ZR, and GA_3_ having the highest average accuracy values. The stomatal area, carotenoid content, *F*_0_, and *C*_i_ exhibited the highest average accuracy values among the stomatal morphology indicators, photosynthetic pigments, chlorophyll fluorescence parameters, and photosynthetic characteristics, respectively (Figure 11b). Furthermore, some indicators showed positive and negative correlations with each other. For example, *C*_i_ exhibited a significant and strong positive correlation with IAA, ZR, and IPA contents (*p* < 0.01) (Figure S1b).

## 3. Discussion

### 3.1. Influence of RpKTI2 Overexpression on Chlorophyll Content and Chlorophyll Fluorescence Characteristics of Tobacco

The overexpression of RpKTI2 from *R. pseudoacacia*, which was highly consistent at the mRNA and protein levels, had a limited influence on chlorophyll synthesis in tobacco. Overexpression of the *Descurainia sophia* TI genes *DsTI1* and *DsTI2* in *Brassica napus* did not significantly influence the chlorophyll content [18], which is consistent with our results. Chlorophyll is essential for the capture and transfer of light energy in plants, and its content is closely related to photosynthetic capacity [19]. Therefore, stability in the chlorophyll content of transgenic plants would to some extent reflect unchanged photosynthetic function. Consequently, the growth and development of the transgenic plants were negligibly enhanced, as indicated by similarities in leaf type (Figure S2), plant height (Figures S3a and S4a) [18,20], biomass [5], ground diameter (Figures S3b and S4b), growth rates of plant height (Figure S5a), plant stem diameter (Figure S5b), and internode length [20,21,22] with WT plants. This indicates that the regulatory contribution of *RpKTI2* to chlorophyll production and plant growth and development was relatively minor, which may be attributed to its minimal effect on endogenous proteins [23].

Chlorophyll fluorescence parameters reflect the light absorption, transfer, and conversion characteristics of plant photosystems, and they are closely related to photosynthesis [24]. *F*_0_ represents the fluorescence radiation intensity when the photosystem II (PSII) reaction centers are fully open [25], and it is negatively correlated with the activity of the reaction centers. In the present study, random forest regression analysis revealed that *F*_0_ had the highest accuracy, suggesting that it was affected by *RpKTI2* overexpression somehow influencing photosynthetic function. *F*_0_ was reduced in the transgenic tobacco, reflecting an increase in the activity of reaction centers and the conversion efficiency of light energy captured by PSII antenna pigments [25]. This is consistent with the observed increase in *F*_v_/*F*_m_. Elevated *F*_v_/*F*_m_ in the *RpKTI2*-positive lines indicates an increase in the light energy participating in photochemical reaction pathways and a reduction in the activation energy consumed through thermal dissipation. This consequently lowers *q*_N_ [26], which leads to enhanced light quantum transport capacity and an increase in *Φ*_PSII_ [27]. This is basically consistent with our experimental results. Therefore, *RpKTI2* overexpression promoted the capture and conversion of light energy via the photosystem in tobacco but had a smaller influence on the activation energy consumption and light quantum transport pathways. As shown in the cluster heatmap (Figure S1a), *F*_0_ and *q*_N_ were separately highly clustered with the ZR, GA3, and ABA contents, indicating that the PSII reaction centers and thermal dissipation pathway may be regulated by endogenous hormones.

### 3.2. Influence of RpKTI2 Overexpression on the Photosynthetic Function and Stomatal Characteristics of Tobacco

*P*_n_ is a direct manifestation of the photosynthetic capacity of plants, which provides organic matter accumulation for plant growth and development [28]. *C*_i_ is closely related to the CO_2_ assimilation rate [29] and provides a direct carbon source for photosynthesis [30], thereby influencing the photosynthetic function of plants. The random forest regression analysis revealed that *C*_i_ had the highest average accuracy among the various photosynthetic characteristics. This suggests that it was most affected by *RpKTI2* overexpression and served as an important factor influencing photosynthetic function. *C*_i_ is influenced by *G*_s_ under normal circumstances. Higher *G_s_* values indicate a bigger quantity of CO_2_ entering the leaves through the stomata, leading to a higher *C*_i_ [31]. Therefore, *C*_i_ is related to stomatal activity, which is in turn controlled by multiple factors, including endogenous hormones that constitute an important regulatory pathway. Specifically, IAA promotes stomatal opening whereas ABA promotes stomatal closure [32]. BR can promote stomatal closure independently of ABA [33], but its effect is dependent on concentration. High concentrations of BR can induce stomatal closure, as observed in *Solanum lycopersicum* [34]. In *RpKTI2*-overexpressing tobacco plants, the IAA content was reduced, whereas the ABA content was relatively stable, and the BR content significantly increased. This resulted in a reduced promoting effect on stomatal opening in the positive transgenic plants, which was consistent with the reductions in stomatal width and area (Table 1). Consequently, the quantity of CO_2_ entering the stomata was reduced, leading to a decrease in *C*_i_.

*RpKTI2* overexpression caused a reduction in the stomatal area and width of leaves in the transgenic lines. This indicates that *RpKTI2* overexpression affected stomatal morphology in tobacco. The decrease in stomatal width and area reduced CO_2_ absorption, thereby affecting the photosynthetic rate of plants. The random forest regression analysis revealed stomatal area had the highest accuracy among stomatal indicators, followed by stomatal length. This indicates that the overexpression of *RpKTI2* exerted a strong influence on stomatal area and stomatal length, highlighting these two indicators as important stomatal factors affecting photosynthetic function [35].

### 3.3. Influence of RpKTI2 Overexpression on Endogenous Hormones of Tobacco

Plant hormones regulate processes such as growth and development, morphogenesis, and stress responses [36]. A previous study found that under moist conditions, the expression of genes encoding protease inhibitors in potatoes was downregulated, whereas the concentrations of endogenous hormones ABA and IAA increased, suggesting a negative correlation [37]. Our results indicated that *RpKTI2*-overexpressing positive transgenic tobacco plants had a significantly lower IAA content than WT plants. The contents of hormones such as ZR, GA_3_, IPA, and ABA decreased in most of the positive transgenic lines and were negatively correlated with expression level, which is in accordance with findings from potatoes [37]. The random forest regression analysis showed that IAA had the highest accuracy. This suggests that the overexpression of *RpKTI2* and RpKTI2 exerted a relatively big effect on IAA. Furthermore, this overexpression is negatively correlated with IAA concentration and might be related to the degradation, transport, or signaling pathways of IAA. However, IAA regulates processes such as embryonic development and organogenesis. Previous research has shown that growth in *Glycyrrhiza uralensis* is positively correlated with IAA concentrations [38,39]. In this experiment, the growth of tobacco plants overexpressing *RpKTI2* was affected to a lesser extent (Figures S2–S5), whereas their root size and morphology were obviously affected (Figure S6). The projection area, surface area, and volume of the root system in *RpKTI2*-overexpressing lines were higher than those of WT lines (Figure S6b,c,e). These changes in the above root indices are essentially negatively correlated with IAA content changes in tobacco, indicating that IAA affects root morphology, and the mode of influence is similar to that of melon radicle and might be related to IAA concentration [40]. This also points to IAA as a possible primary effect of *RpKTI2*-overexpression and WT tobacco, when compared to other indices.

In the present study, the BR content of the transgenic tobacco increased and was positively correlated with *RpKTI2* expression. BRs are polyhydroxylated steroid hormones that enhance plant resistance to stresses through the BR signaling pathway and its regulatory network [41]. These hormones induce the accumulation of ethylene (ET) by inducing the production of H_2_O_2_. Both ET and H_2_O_2_ function as signaling molecules that can activate intracellular antioxidant pathways, thereby attenuating oxidative damage [42,43]. TIs can regulate endogenous protein activity in animals and plants, possessing strong antioxidant capability [44,45]. When the larvae of *Callosobruchus chinensis* were reared on mung beans containing TIs for four consecutive generations, their antioxidant enzymes, such as superoxide dismutase and catalase, were significantly inhibited [44]. However, trypsin isolated and purified from *Annona squamosa* seeds showed considerable free radical scavenging activity. An IC50 value of 108 μg/mL indicates the presence of strong antioxidant properties [45]. Therefore, the *RpKTI2* overexpression increases the antioxidant capacity of positive transgenic tobacco lines and enhances their resistance to biotic and abiotic stresses.

## 4. Materials and Methods

### 4.1. Materials

Tobacco (*Nicotiana tabacum*, cultivar NC89) seeds and an *Agrobacterium* solution that had been transformed with the pBI121-*RpKTI2* plasmid were supplied by our laboratory.

### 4.2. Cultivation and Identification of Transgenic Tobacco Plants Expressing RpKTI2

Tobacco seeds were disinfected with 70% alcohol for 30 s, washed with sterile water, soaked in 1% NaClO for 10 min, rinsed 4–5 times with sterile water, patted dry, inoculated onto a 1/2 Murashige & Skoog (MS) medium, and cultured for 30 d to obtain sterile seedlings. Leaves were subjected to removal of the main vein and margins, cut into 1 cm^2^ pieces, inoculated onto a co-culture medium (solid MS medium + 2.0 mg·L^−1^ 6-BA + 0.5 mg·L^−1^ NAA), and cultured in the dark for 2 d. Subsequently, the leaf pieces were placed in *RpKTI2*-transformed *Agrobacterium* bacterial solution (OD_600_ = 0.6–0.8) for impregnation for 10–15 min, and cultured on co-culture medium in the dark for another 2 d. After culturing, the leaf pieces were rinsed with sterile water, patted dry, and placed on a selection medium (solid MS medium + 2.0 mg·L^−1^ 6-BA + 0.5 mg·L^−1^ NAA + 50 mg·L^−1^ Kan + 400 mg·L^−1^ Cef) to induce shoot differentiation. When the shoots grew to a height of 2–3 cm, they were transferred onto rooting medium (solid MS medium + 0.1 mg·L^−1^ NAA + 50 mg·L^−1^ Kan + 400 mg·L^−1^ Cef) to induce rooting. Leaf DNA was extracted and polymerase chain reaction (PCR) was performed with specific primers (Table 2) for initial identification of positive transgenic plants. The binding site of the forward-specific primers is the 1st–24th bases, while that of the reverse-specific primers is the 610th–633rd bases of the *RpKTI2* Coding sequence (CDS) region.

The positive transgenic plants were further cultivated until the T0 generation seeds were harvested. T0 generation seeds were cultured on a screening medium containing 350 mg·L^−1^ Kan to obtain the T1 generation tobacco plants. Upon the emergence of two true leaves, the T1 generation tobacco plants were transplanted into potting soil (peat:vermiculite = 3:1) for indoor domestication. The domestication conditions were as follows: 16 h light/8 h dark cycle, 25 °C, 60–80% relative humidity, and light intensity of 2000–3000 lx. Real-time quantitative reverse transcription PCR (RT-qPCR) was performed on the T1 generation transgenic tobacco with *NtActin* used as the housekeeping gene. Fold changes were expressed relative to WT, and three plants in each line were chosen for RT-qPCR. Relative gene expression levels were calculated using the 2^−ΔΔCt^ method, and all experiments were repeated three times.

### 4.3. Extraction and Quantitative Analysis of RpKTI2

Fresh plant leaves were taken from transgenic and WT plants and then placed in a precooled mortar at 3 °C. Liquid nitrogen was added, and the leaves were ground into powder. Then, 100 mg of powder was weighed and placed into a 1.5 mL centrifuge tube. A Plant Total Protein Extraction Kit (Sangon Biotech (Shanghai, China) Co., Ltd., Shanghai, China) was used to extract the total protein from the powder.

The extracted protein was dissolved in 1 mol/L Tris HCl (pH 8.0) and centrifuged at 15,000 r·min^−1^ at 4 °C for 5 min. The supernatant was then mixed with SDS-PAGE Loading Buffer, and SDS-PAGE electrophoresis was performed. The electrophoresis gel plate was stained with 1 mg/mL Coomassie Brilliant Blue G-250 range for 1.5 h and then decolorized with the decolorization solution for 10 h. Then, the electrophoretic gel tape containing the target protein was cut off and placed in a 5 mL centrifuge tube. Micro Protein PAGE Recovery Kit (Sangon Biotech (Shanghai) Co., Ltd., Shanghai, China) was used to recover the target protein.

The recovered target protein was dissolved in 1 mol/L Tris HCl (pH 8.0). The supernatant (40 μL) was mixed with 200 μL 0.1 mg·mL^−1^ Coomassie Brilliant Blue G-250, then stewed for 2 min. SpectraMax 190 Enzyme-linked immunosorbent assay (ELISA) detector (Molecular Devices, San Jose, CA, USA) was used to measure the OD value of the target protein at 595 nm. The target protein content in mixed liquid was obtained from the standard curve, and the concentration of the target protein in plant tissue was calculated in this experiment. Testing was performed in triplicate.

### 4.4. Measurement of Chlorophyll Content

Chlorophyll content was measured using the acetone-anhydrous ethanol extraction method. The second leaf of the upper section was obtained from both the transgenic and WT plants. A 0.3 g sample of the fresh leaf was cut into small pieces and immersed in 10 mL of acetone: anhydrous ethanol solution (2:1, *v*/*v*) at room temperature in the dark for 12 h, at which point the leaf tissue was completely white. Absorbance values of the extract solution were measured using a TU-1810 UV–Vis spectrophotometer (440, 644, and 662 nm; Shanghai, China), with measurements performed in triplicate. The various indicators were calculated using the following formulae:

Chlorophyll a (Chla) content = 9.78 × A662 − 0.99 × A644 (1)

Chlorophyll b (Chlb) content = 21.43 × A644 − 4.65 × A662(2)

Total chlorophyll (Chla + b) content = 5.13 × A662 + 20.44 × A644 (3)

Carotenoid content = 4.70 × A440 − 0.27 × Chla + b(4)

where A440, A644, and A662 are the absorbance values of the mixed extract solution at 440, 644, and 662 nm, respectively.

### 4.5. Chlorophyll Fluorescence Parameter Measurements

Chlorophyll pulse transient fluorescence kinetic indicators of transgenic tobacco lines were measured using a Yaxin-1161G handheld chlorophyll fluorometer (Yaxin, Beijing, China) in the nursery room. Fully expanded leaves were selected from the middle section of plants in each group. Leaves were clamped with leaf clips and subjected to dark treatment for 20 min, followed by a saturating light pulse treatment at 3000 μmol·m^−2^·s^−1^ for 1 s and actinic light treatment at 1000 μmol·m^−2^·s^−1^ for 9 s. The light energy was provided by the built-in light source of the chlorophyll fluorescence meter. The initial fluorescence (*F*_0_), maximum photochemical efficiency (*F*_v_/*F*_m_), non-photochemical quenching coefficient (*q*_N_), and actual photochemical quantum efficiency (*Φ*_PSII_) were measured to evaluate the PSII reaction center activity, maximum photosynthesis efficiency, thermal dissipation of stimulated light energy, and PSII electron transport status of the tobacco lines. Measurements were performed in quadruplicate for each group.

### 4.6. Measurement of Photosynthetic Function

Tobacco plants in the nursery room were subjected to photosynthetic rate (*P*_n_), stomatal conductance (*G*_s_), intercellular CO_2_ concentration (*C*_i_), and transpiration rate (*T*_r_) measurements using an ECA-PB0402 photosynthesis analyzer (Yinongkang, Beijing, China) with single leaf closed circuit mode. The leaf chamber was opened, and the handle was gently swung until the CO_2_ concentration stabilized. Fully expanded leaves were obtained from the middle and upper sections of plants and securely clamped in the leaf chamber. The measurement process only commenced after the CO_2_ value had decreased steadily. During the testing course, the indoor temperature was 24.5 °C, and the illuminance was 3000 lx (approximately 38.17 μmol·m^−2^·s^−1^ of light quanta intensity), derived from the incandescent tubes on the cultivation rack. Measurements were performed in quadruplicate for each group.

### 4.7. Observation of Stomatal Morphology

The central portion of each leaf sample was removed, cut into 2 × 2 cm^2^ pieces, and attached to transparent tape, with the underside of the leaf facing upwards. Another piece of transparent tape was adhered to the back of the leaf pieces and the lower epidermis was slowly peeled off for observation and photography under an Olympus DP22 microscope (Olympus, Tokyo, Japan). The procedure was performed in triplicate for each group, and three different fields of view were photographed during each repetition. The stomatal length, width, area, and density of the leaves were measured using ImageJ 1.8.0 (LOCI, Bethesda, MD, USA), with measurements performed thrice for each image.

### 4.8. Measurement of Endogenous Hormone Content

Plant hormone concentrations, including gibberellin (GA3), indole-3-acetic acid (IAA), indole-3-propionic acid (IPA), zeatin riboside (ZR), abscisic acid (ABA), brassinosteroid (BR), and others, were measured using enzyme-linked immunosorbent assay [46,47,48]. The third leaf of the upper section was obtained from the tobacco plants, and a 0.2 g sample was weighed, placed in a pre-chilled mortar, and ground into powder in the presence of liquid nitrogen. The powder was placed in a 5 mL centrifuge tube. The powder was transferred to a centrifuge tube, and 2 mL of sample extraction solution (80% methanol, containing 1 mmol·L^−1^ BHT (di-*tert*-butyl-*p*-cresol)) was added to clean the mortar. The extraction solution and residue were transferred to a test tube and shaken well. Subsequently, the mixture was placed in a refrigerator and extracted at 4 °C for 4 h, followed by centrifugation at 3500 r·min^−1^ for 8 min. Finally, the supernatant was removed. Subsequently, 1 mL of extraction solution was added to the precipitate, and the mixture was shaken well, extracted at 4 °C for 1 h, centrifuged at 3500 r·min^−1^ for 8 min, and the supernatant was merged. The supernatant was passed through a C-18 solid-phase extraction column to extract hormone samples. The samples were transferred to a 10 mL centrifuge tube, concentrated and dried under vacuum, and then dissolved with the sample diluent (500 mL PBS containing 0.5 mL Tween-20 and 0.5 g gelatin that was slightly heated and dissolved), which was the endogenous hormone extraction solution.

An appropriate amount of Sigma standard sample diluent (St. Louis, MO, USA) was taken, and eight different concentrations of standard sample solutions (including 0 ng·mL^−1^) were prepared to make standard curves. However, the maximum concentrations of IAA and ABA were 50 ng·mL^−1^ and those of BR, GA3, and ZR were 10 ng·mL^−1^. An appropriate volume of antibody was added to 5 mL of sample diluent according to the appropriate dilution ratio, mixed well, and then the mixture was added to the ELISA plate, with a volume of 50 μL per pore. The ELISA plate was then placed in a wet box for competition and reacted for 0.5 h at 37 °C. Subsequently, the reaction solution was shaken dry, the water stains were removed, and the washing solution (1000 mL PBS plus 1 mL Tween-20) was added to the ELISA plate. After being completely shaken dry, the washing solution was added for the second time, and the plate was washed four times repeatedly. Similarly, an appropriate volume of ELISA secondary antibody was added to 10 mL of sample diluent according to the appropriate dilution ratio, mixed well, and 100 μL was added per pore. The subsequent competition reaction and washing of the ELISA plate were the same as before. o-phenylenediamine (10–20 mg) was added and mixed with 10 mL of substrate buffer (5.10 g C_6_H_8_O_7_·H_2_O, 18.43 g Na_2_HPO_4_·12H_2_O, 1000 mL distilled water, and 1 mL Tween-20, pH 5.0). After full dissolution, 4 μL of 30% H_2_O_2_ was added to the mixture and shaken well to prepare the chromogenic solution. The ELISA plate was then placed in a wet box for chromogenic reaction after the addition of 100 μL chromogenic solution per pore. After appropriate color development, 50 μL of 2 mol·L^−1^ H_2_SO_4_ was added to terminate the reaction. Finally, a SpectraMax 190 ELISA detector (Molecular Devices, San Jose, CA, USA) was used to measure the OD values of the standard sample and each sample at 490 nm. Testing was performed in triplicate.

ELISA results were calculated by logistic curves. The horizontal axis of the curve represents the natural logarithm of each concentration (ng∙mL^−1^) of the hormone standard, and the vertical axis represents the logistic value of each concentration’s colorimetric value. The calculation method for the Logit value is as follows:

LogitBB0=ln⁡BB01−BB0=ln⁡(BB0−B)

where B_0_ is the colorimetric value of 0 ng·mL^−1^ pore and B is the colorimetric value of the sample pore.

### 4.9. Data Processing

Data were statistically analyzed in SPSS 27.0 (SPSS, Chicago, IL, USA). One-way analysis of variance (ANOVA) was conducted at a significance level (α) of 0.05, and multiple comparisons were performed using Duncan’s new multiple-range test. Graphs were plotted in the Origin 2018 software (Origin Lab, Northampton, MA, USA). Principal component analysis (PCA) and random forest analysis were performed using MetaboAnalyst 5.0 (Xia-Lab, McGill University, Montreal, QC, Canada).

## 5. Conclusions

Tobacco lines overexpressing *RpKTI2* from *R*. *pseudoacacia* exhibited relatively small changes in chlorophyll content, chlorophyll fluorescence characteristics, and photosynthetic function, with only *F*_v_/*F*_m_ showing a significant increase and *C*_i_ showing a significant decrease. Stomatal morphology was altered, with stomatal length and area exhibiting overall decreases. IAA, ZR, GA_3_, and IPA contents were lowered, leading to a weaker regulation of plant growth and development, whereas the BR content increased. This enhanced the antioxidant capacity of the plants. The characteristics of these transgenic tobacco lines may also have been partially influenced by kanamycin selection in the experiment. In conclusion, *RpKTI2* overexpression exerted a relatively small influence on chlorophyll synthesis and photosynthetic system characteristics while having a considerable impact on stomatal morphology, photosynthetic function maintenance, leaf type and growth, and antioxidant capacity. The current findings provide a basis and reference for future in-depth research on *RpKTI2*, contributing to new lead-tolerance plant germplasm and genetic resources useful in lead-contaminated soil.

## Figures and Tables

**Table 1:** Variation of stomatal morphology of transgenic *Nicotiana tabacum.* The vertical bar in the table represents the standard error (*n* = 3), and the different lowercase letters indicate significant differences between the lines (*p* < 0.05).

Lines	Length/μm	Width/μm	Area/μm^2^	Density/mm^2^
WT	22.22 ± 0.26 ab	10.11 ± 0.76 ab	162.00 ± 14.60 ab	108.78 ± 10.19 bc
K1	18.24 ± 0.45 c	9.80 ± 0.22 bc	122.61 ± 6.28 c	169.39 ± 6.77 a
K2	22.92 ± 1.02 a	11.08 ± 0.14 a	190.65 ± 14.17 a	101.01 ± 4.11 c
K3	16.37 ± 0.70 c	8.89 ± 0.51 bc	116.28 ± 12.15 c	144.52 ± 13.99 a
K4	17.43 ± 0.55 c	8.60 ± 0.21 c	122.99 ± 9.16 c	136.75 ± 6.22 ab
K5	20.63 ± 0.51 b	9.46 ± 0.14 bc	166.93 ± 7.12 ab	104.12 ± 13.28 c
K8	18.26 ± 0.07 c	9.64 ± 0.25 bc	138.58 ± 6.85 bc	163.17 ± 11.73 a

**Table 2:** Primers used in this experiment.

Function	Primer Name	Sequence (5′–3′)
Identification of *RpKTI2* transgenic *Nicotiana tabacum* positive seedlings	OE-*RpKTI2*	F: GAGAACACGGGGGACTCTAGAATGAAGCCTGCATTTATTACCCTC R: CGATCGGGGAAATTCGAGCTCTCAAACTACTGAGTTTCCAGTGGC
RT-qPCR	NtActin	F: ACCTCTATGGCAACATTGTGCTCAG R: CTGGGAGCCAAAGCGGTGATT
	q*RpKTI2*	F: CCAAGTGGGTAGTGGTTGCT R: ACTGAGTTTCCAGTGGCGTC

## Data Availability

The main results and Supplementary Data have already been presented in the manuscript, and the original data can be obtained from the corresponding author upon reasonable request.

## Supplementary Materials

The following supporting information can be downloaded at https://www.mdpi.com/article/10.3390/plants13131867/s1, Figure S1: (a) Correlation analysis and (b) heat map between various indicators of tobacco lines. The different colors represent the value of the coefficient. A value above zero represents positively correlated and value below zero represents negatively correlated; Figure S2: Leaf morphology of transgenic *Nicotiana tabacum:* (a) length of blade, (b) blade width, (c) leaf area, (d) length/width; Figure S3: Growth characteristics of transgenic *Nicotiana tabacum* in the previous stage (29 February 2024)*:* (a) plant height and (b) ground diameter; Figure S4: Growth characteristics of transgenic *Nicotiana tabacum* in the later stage (22 May 2024): (a) plant height and (b) ground diameter; Figure S5: Growth rate of transgenic *Nicotiana tabacum* (a) seedling height and (b) seedling stem diameter; Figure S6: Root morphology and characteristics of transgenic *Nicotiana tabacum:* (a) total root length, (b) total root projection area, (c) total root surface area, (d) average root diameter, (e) total root volume, (f) root tip number; Table S1: Full-length transcript sequence of *RpKTI2*; Table S2: Encoding sequence of *RpKTI2*; Table S3: Amino acid sequence of RpKTI2.
