# Supplementary material for: Overexpression of RpKTI2 from Robinia pseudoacacia Affects the Photosynthetic Physiology and Endogenous Hormones of Tobacco
Source: Plants (Basel). 2024 Jul 6;13(13):1867. doi: 10.3390/plants13131867 (PMC11243900; doi:10.3390/plants13131867)
Supplement: Supplementary file 1 [file plants-13-01867-s001.zip › plants-3060517-supplementary.pdf]

Supplementary Table S1. The full-length transcript sequence of *RpKTI2* gene.

**Base sequence**
ATGGGGAGAGAGAAACCAGCATATCCTTGACAGACCATGAAGCCTGCATTTATTACCCTCTCTTTCCTACTTTTTGCCTTCA
TCACTAACCTTCAATTAACCTTTTCACAAGATGTTGAACAAGTGGTGGATACAAATGGAAACCCCATTTTTCCTGGTGGCAG
ATTCTATATTTTGCCAGCTATCTCTGGCCCAGCAGGAGGTGGAGTAAGACTTGGCAAAACTGGGAACTCAACATGCCCAGTT
ACTGTGCTGCAAGATTATTCTGAGGTTATCAATGGCTTGCCAGTAAAATTCAGCATACCTGGAATAAGCCCTGGTATCATTT
TTACAGGCACATCACTAGATATTGCATTTGAAGAGAAGCCTGAGTGTGCTGGATCATCCAAGTGGGTAGTGGTTGCTGATGA
TTTCCCAAGAGAATGGGTGGGTATTGGTGGTGAAGAAGACCATCCAAGCAAGCAAATCCTAGAAGGTAGGTTTAACATCAGA
AATATGGTTCGGGTTACAAGCTTGTGTTCTGTCCCACAATCACCGCTCCACCTGGTGTTTGTTTTGATATTGGGAGGTACAA
TGATGAGAATGGAAGGCGTCTGATCCTCACTGAGGATGATCCCTATGAAGTTGTTTTCATTGATGCTGACGCCACTGGAAAC
TCAGTAGTTTGAATTGAATCCCCACACCGTTGAATGATAAATGGTGTAGTAATAGTTCTTCTTATGATGTATTATCATCCTG
CACTAATCAATGTAAGATACTTTTTAACACAAGAAAAAAAAAAAAAAAAAAAAAAAAA

Supplementary Table S2. The encoding sequence of *RpKTI2* gene.

**Base sequence**
ATGAAGCCTGCATTTATTACCCTCTCTTTCCTACTTTTTGCCTTCATCACTAACCTTCAATTAACCTTTTCACAAGATGTTG
AACAAGTGGTGGATACAAATGGAAACCCCATTTTTCCTGGTGGCAGATTCTATATTTTGCCAGCTATCTTTGGCCCAGCAGG
AGGTGGAGTAAGACTTGGCAAAACTGGGAACTCAACATGCCCAGTTACTGTGCTGCAAGATTATTCTGAGGTTATCAATGGC
TTGCCAGTAAAATTCAGCATACCTGGAATAAGCCCTGGTATCATTTTTACAGGCACATCACTAGATATTGCATTTGAAGAGA
AGCCTGAGTGTGCTGGATCATCCAAGTGGGTAGTGGTTGCTGATGATTTCCCAAGAGAATGGGTGGGTATTGGTGGTGAAGA
AGACCATCCAAGCAAGCAAATCCTAGAAGGTAGGTTTAACATTCAGAAATATGGTTCGGGTTACAAGCTTGTGTTCTGTCCC
ACAATCACTGCTCCACCTGGTGTTTGTTTTGATATTGGGAGGTACAATGATGAGAATGGAAGGCGTCTGATCCTCACTGAGG
ATGATCCCTATGAAGTTGTTTTCATTGATGCTGATGCCACTGGAAACTCAGTAGTTTGA

Supplementary Table S3. The amino acid sequence of RpKTI2 protein.

**Amino acid sequence**
MKPAFITLSFLLFAFITNLQLTFSQDVEQVVDTNGNPIFPGGRFYILPAIFGPAGGGVRLGKTGN
STCPVTVLQDYSEVINGLPVKFSIPGISPGIIFTGTSLDIAFEEKPECAGSSKWVVVADDFPREW
VGIGGEEDHPSKQILEGRFNIQKYGSGYKLVFCPTITAPPGVCFDIGRYNDENGRRLILTEDDPY
EVVFIDADATGNSVV

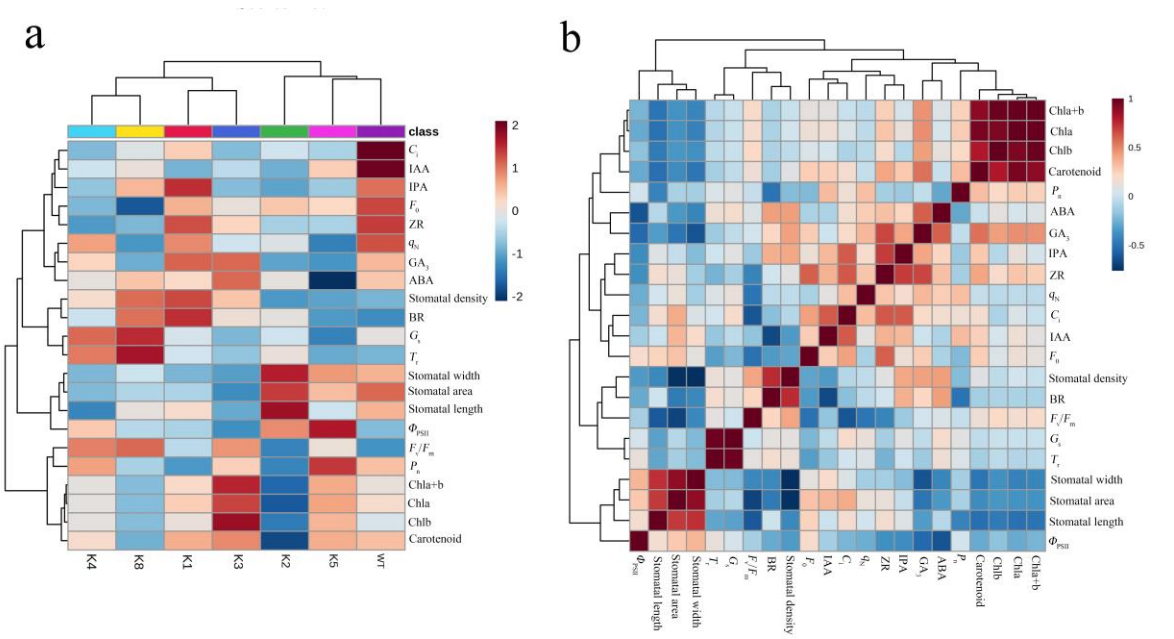

Figure S1. (a) Correlation analysis and (b) heat map between various indicators of tobacco lines. The different colors represent the value of the coefficient. A value above zero represents positively correlated and value below zero represents negatively correlated.

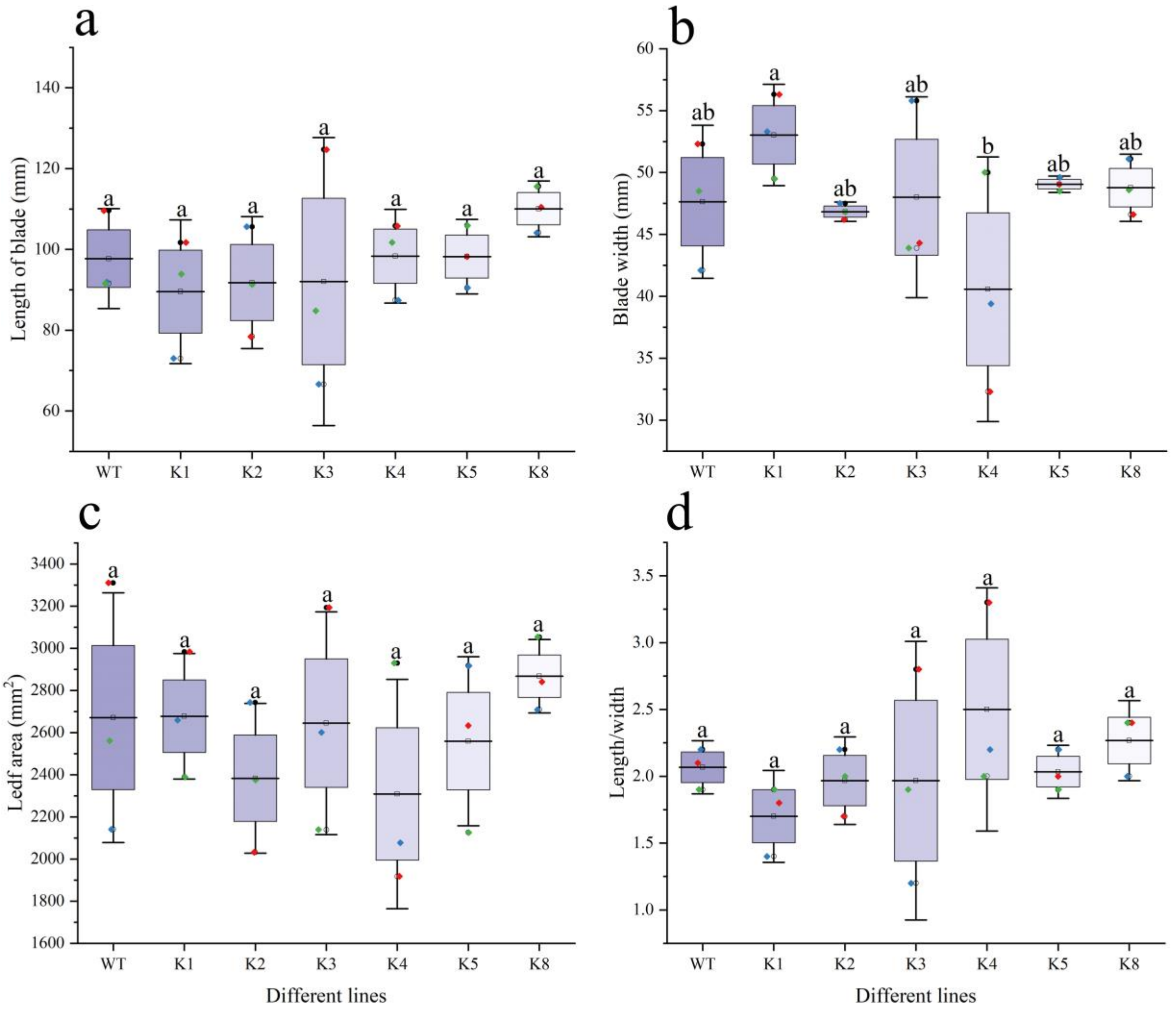

Figure S2: Leaf morphology of transgenic *Nicotiana tabacum:* (a) length of blade, (b) blade width, (c) leaf area, (d) length/width.

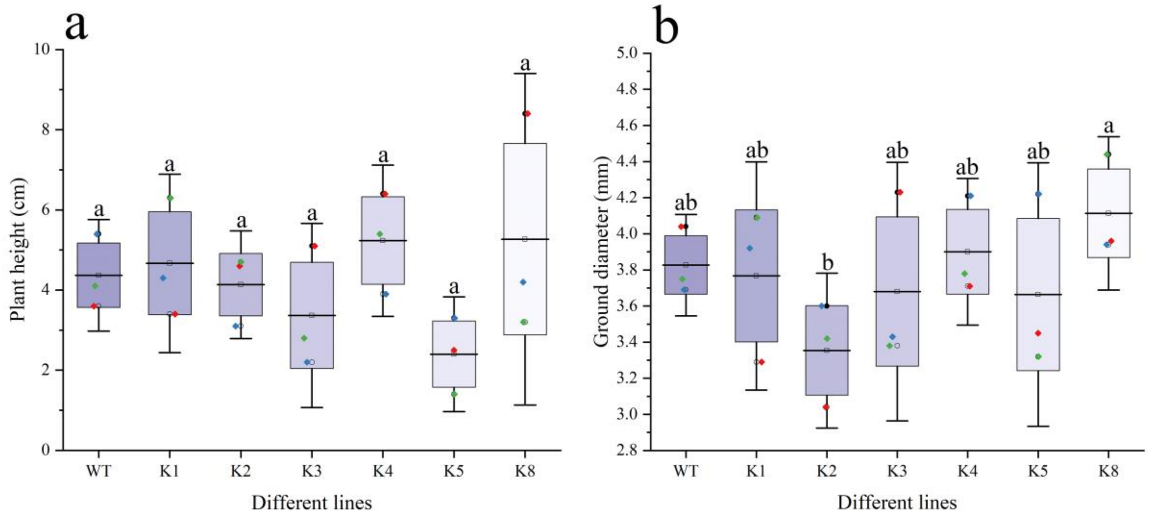

Figure S3: Growth characteristics of transgenic *Nicotiana tabacum* in the previous stage (29 February 2024)*:* (a) plant height and (b) ground diameter.

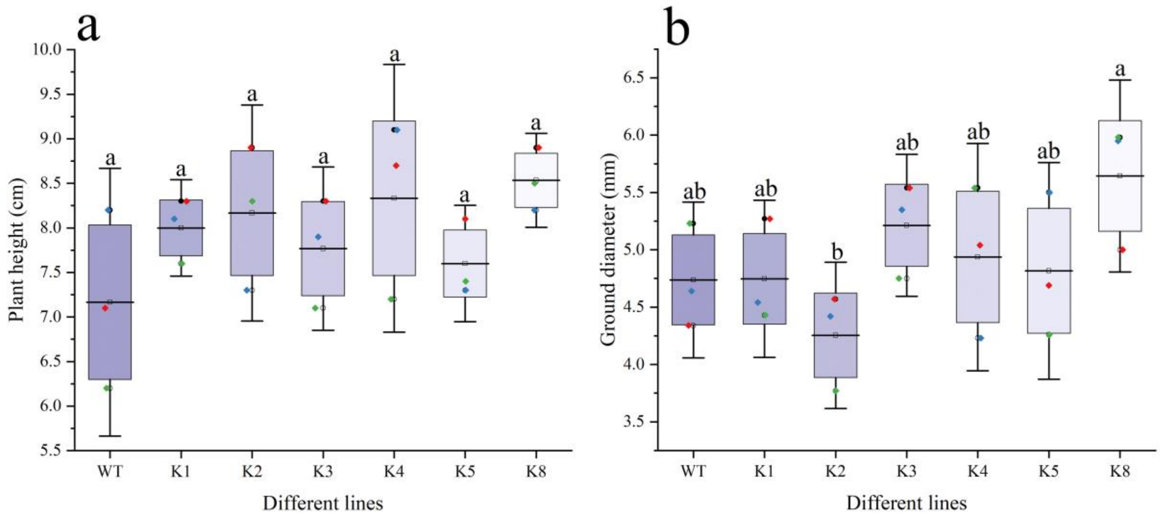

Figure S4: Growth characteristics of transgenic *Nicotiana tabacum* in the later stage (22 May 2024): (a) plant height and (b) ground diameter.

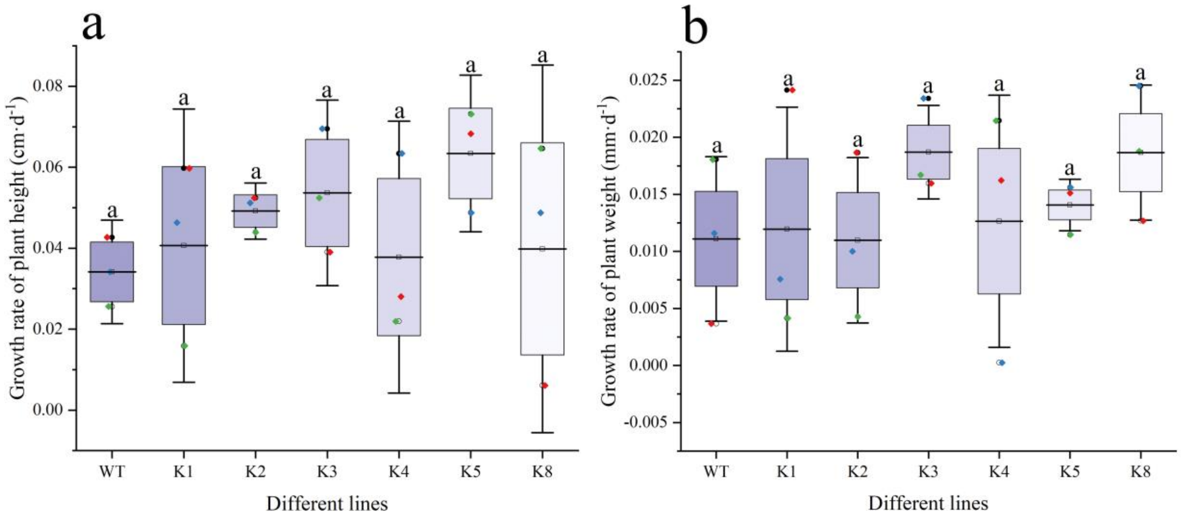

Figure S5: Growth rate of transgenic *Nicotiana tabacum* (a) seedling height and (b) seedling stem diameter.

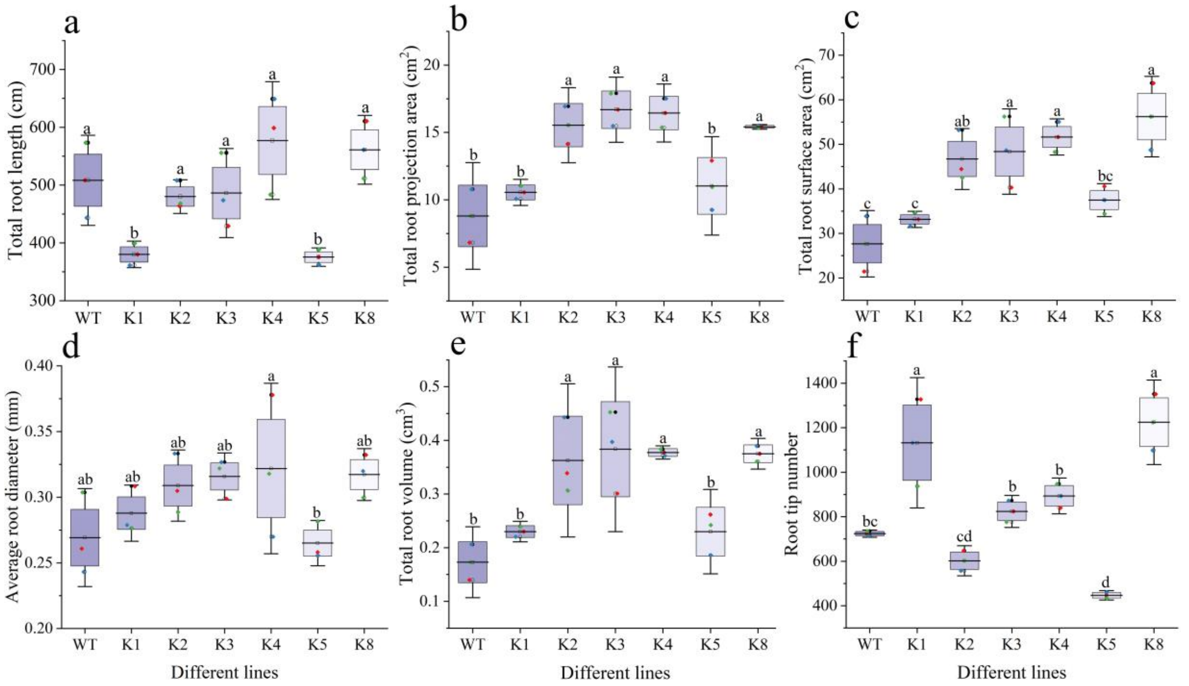

Figure S6: Root morphology and characteristics of transgenic *Nicotiana tabacum:* (a) total root length, (b) total root projection area, (c) total root surface area, (d) average root diameter, (e) total root volume, (f) root tip number.
